# Effects of old age on fatigability and sensorimotor characteristics of a repetitive upper limb fatiguing task

**DOI:** 10.1371/journal.pone.0235314

**Published:** 2020-07-09

**Authors:** Christopher A. Bailey, Maxana Weiss, Julie N. Côté

**Affiliations:** 1 Department of Kinesiology and Physical Education, McGill University, Montreal, Quebec, Canada; 2 Centre for Interdisciplinary Research in Rehabilitation, Jewish Rehabilitation Hospital Research Center, Laval, Quebec, Canada; Washington University in Saint Louis School of Medicine, UNITED STATES

## Abstract

**Objectives:**

1) Determine the effects of old age on sensorimotor responses to a fatiguing work-like task. 2) Explore how old age influences the relationships between task fatigability, everyday perceptions of fatigability, and sensorimotor function.

**Methods:**

Healthy young (N = 17, 9W) and older (N = 13, 10W) adults completed the Pittsburgh Fatigability Scale to assess everyday perceptions of physical (PF) and mental fatigability and performed a repetitive tapping task to fatigue. Before and after the task, grip strength was assessed using a hand-grip dynamometer and touch-pressure sensitivity was measured (shoulder, hand) using Semmes-Weinstein monofilaments.

**Results:**

Older, but not young adults, had increased touch-pressure sensitivity at the shoulder after fatigue (interaction, p = 0.007). No changes in grip strength were observed (p>0.05). Task fatigability was not different between young and old adults (p>0.05). Having less task fatigability was associated with lower PF, higher grip strength, and higher touch-pressure sensitivity at the hand (ρ = 0.37–0.58, p<0.05), with the hand sensation association also observed in the old adult subgroup (ρ = 0.56, p = 0.046).

**Conclusions:**

With old age, there were fatigue-related alterations to sensory but not physical function. While task fatigability was associated with perceptual, physical, and sensory features, sensory features appear to have a more important role with old age.

## Introduction

Work-related musculoskeletal disorders (WMSDs) are the single largest cause of absenteeism and work disability [1] and have a negative impact on quality of life and efficacy of the workforce, and on the economy. Surveys of the working population suggest that 20–30% of workers have experienced at least one WMSD [1]. The term ‘WMSD’ is used to categorize the development of disorders affecting joints, soft tissue, and connective tissue [1] with an unknown cause, but for which work-related exposures such as fatigue are a factor [2].

WMSDs have been linked to repetitive movement with fatigue, high force, and poor posture, as well as personal factors such as worker’s age as risk factors [1,3,4]. Moreover, population dynamics are shifting, with the number of children aged 0–14 years remaining constant and the number of seniors (adults aged 65+ years) increasing by nearly 3% from the 2011 to the 2016 Canadian Census [5]. One in five Canadian seniors reported working throughout the 2015 year, the highest proportion since the 1981 census, with more than 30% of senior workers engaged in full-time work [6]. Thus, the workforce does not actually end at the expected age of 65 years; understanding how young and old adults respond to repetitive motion-induced fatigue and the functional factors that reduce fatigability is of importance.

Aging results in physiological changes to muscles and the neuromuscular system, reducing muscle strength and affecting endurance [7]. As aging progresses, decreases in function affect strength, balance, and gait control, resulting in increased risk of injury [8–11]. There is no singular cause for the aging-related changes in muscle structure and function. Insights into physical functioning, however, can be gained by investigating grip strength. Grip strength is a clinically significant measure of physical function relevant to activities of daily living such as grasping [12]. Grip strength has been shown to be a predictor of age-related disability, with older adults displaying less grip strength compared to younger adults [12].

Additionally, sensory system measures have been shown to decline with old age [13] as well as with fatigue [14]. Quantitative sensory testing (QST) modalities measuring threshold to thermal pain, pressure pain, vibration and touch, have been utilized to identify sensory impairment and recovery from peripheral nerve injury [15], where higher sensory thresholds are indicative of poorer sensation. However, to our knowledge, no studies have assessed whether fatigue further impairs the sensory system of older adults.

Finally, fatigue-related changes in physical and sensory function may be exacerbated by everyday perceptions of fatigability, as fatigue is a symptom of both perceived and task-related characteristics [16]. Old adults who possess higher everyday perceptions of fatigability have been shown to have lower physical function and fitness, and, importantly, higher fatigability in standardized walking tasks [17,18]. Unfortunately, it is unclear how everyday perceptions of fatigability relate to upper limb fatigability, and what relationships may exist with resting sensorimotor function. Combined with an understanding of the sensorimotor responses to upper limb fatigue in young and old adults, this information could help predict the likelihood of fatiguing work representing an elevated injury risk in the aging population and could help gain a better grasp of the mechanisms underlying these potential changes.

In summary, there is a knowledge gap about how old age affects the sensorimotor response to fatigue. Elucidating this gap could help develop strategies to optimize participation of older adults to fatiguing work. The purposes of this study were to 1) determine the effect of old age on upper limb physical and sensory function before and after a fatiguing work-like task, and 2) explore the influence of old age on relationships between task fatigability, perceptions of everyday fatigability, and resting sensorimotor function in a fatiguing task with comparable fatigability between old and young adults [19]. We hypothesized that grip strength would decrease and sensory thresholds would increase more in old than in young adults. We also hypothesized that task fatigability would be related to different resting features of everyday perceived fatigability and sensorimotor function in old compared to young adults.

## Materials and methods

### Participants

Convenience samples of asymptomatic young (20–29 years old) and older (> 55 years old) adults were recruited from local university and community populations. Participants were excluded if they self-reported a history of pain or diagnosed musculoskeletal conditions affecting the shoulder or the neck region, or any neurological condition. Participants recruited reported no chronic pain conditions, no pain at present (with individual confirmations for the shoulder, back, and upper extremity region), and had not taken any pain medicine within the previous 24 hours. Two participants, one young male participant and one older male participant, previously had lower limb surgery, i.e. knee surgery; they were medically cleared and pain free at the time of data collection and were cleared to complete the study. Ethical approval for this project was obtained from the Center for Interdisciplinary Research in Rehabilitation (CRIR-504-0410) and written and informed consent was obtained from each individual participant.

### Measurements

#### Anthropometrics

Anthropometric measures of height, mass, body mass index (BMI), and functional arm reach were documented. Height was measured using measuring tape and with the participant standing upright with their head, scapula, and pelvis contacting a wall. A clipboard was placed on top of the head parallel to the floor, and following the participant expiring a deep breath, height was measured as the vertical distance from the floor to the clipboard. Mass was measured using a digital scale, and BMI was quantified as a ratio between height and mass (kg/m^2^). Functional arm reach was measured for the dominant arm as the distance from the acromion to the middle fingertip at 90 degrees of shoulder flexion, full elbow extension, and with the hand and fingers pointing forward.

#### Everyday perceptions of fatigability

The participant then completed the Pittsburgh Fatigability Scale (PFS) to determine general perceptions of both physical and mental fatigability in daily life situations [17]. The participant was instructed to report the “physical and mental fatigue you expect or imagine you would feel immediately after completing each … activity”. Physical and mental fatigability scores corresponding to each of ten activities are independently scored on a 6-point scale from 0 (no fatigue) to 5 (extreme fatigue) and then summed. Summed physical fatigability (PF) and mental fatigability (MF) scores range from 0 to 50, with higher scores indicating higher fatigability. Previous research has shown that PF scores of 15 or higher and MF scores of 13 or higher indicate elevated physical and mental fatigability respectively [18]. This questionnaire has been validated for individuals aged 60 and older [17]; however, it was administered to all participants for consistency.

#### Touch-pressure sensitivity

The participant sat in front of a height-adjustable table set 5 mm below their elbow height, with their hips, knees and ankles flexed 90 degrees. Here, pre-fatigue measures of touch-pressure sensitivity were taken. Touch pressure sensitivity was evaluated using the Semmes-Weinstein filament method of Quantitative Sensory Testing (QST; Touch-Test^™^ Sensory Evaluator, North Coast Medical Inc.). With the participant’s eyes closed, a series of filaments were applied perpendicularly to the skin, in an ascending order of filament thickness proportional to stiffness, until the first one is detected before it bends [20]. Filament thicknesses scores are reported on a logarithmic scale ranging from 1.65–6.65 that corresponds to applied forces from 0.008–300 g, where a sensory threshold at a higher filament thickness is indicative of lower touch-pressure sensitivity [20]. Touch-pressure sensitivity measurements were taken at both the anterior deltoid (QST Shoulder) and the palmar aspect of the hand (QST Hand), indicative of shoulder and hand touch-pressure sensitivity, respectively.

#### Grip strength

Grip strength was measured following touch-pressure sensitivity measurements. The participant completed three trials of a maximal grip strength test using a hand-grip dynamometer (Model #12–0455, DynEx). With verbal encouragement, they were to ramp up (2s), reach and maintain maximal force (3s), and ramp down (2s), with 30s between each trial. The mean of the three trials was recorded as the participant’s grip strength, following previously established protocols [21].

### Fatiguing task procedure

The participant then completed the fatiguing task which consisted of a repetitive tapping motion between two points placed on the table (30% and 100% of functional arm reach) from the seated position at a frequency of 2 Hz (one reach every 0.5 s) as kept by an audible metronome. Each minute, participants were asked to report their rating of perceived exertion (RPE) on the BORG CR-10 scale [22] for the neck/shoulder region. The repetitive task continued until participants reached a RPE ≥ 8 or a task duration of 45 minutes. Participants were unaware of these stoppage criteria. Task fatigability measures recorded included time to task termination and time to RPE of 5 (corresponding to half of the scale, or a verbal descriptor of “hard (heavy)”[22]). Immediately following task termination, post-fatigue task measurements were taken for grip strength, QST Shoulder, and QST Hand.

### Statistical analysis

Outcomes analyzed included anthropometrics (height, mass, BMI), everyday perceptions of fatigability (PF, MF), task fatigability (time to task termination, time to RPE of 5), grip strength, and touch-pressure sensitivity (QST Shoulder, QST Hand). Using SPSS (V24, IBM), we conducted one-way ANCOVAs (with gender as a covariate) to test for statistically significant effects of Age (young, old) on measures of anthropometrics, everyday perceptions of fatigability, and task fatigability. We conducted repeated measures ANCOVAs (with gender as a covariate) to test for statistically significant effects of Age (young, older), Condition (pre-fatigue, post-fatigue), and interactions of Age x Condition on measures of grip strength and touch-pressure sensitivity. Interaction effects were investigated post-hoc using Bonferonni pairwise comparisons. Partial eta squared coefficients (η^2^) were computed to evaluate ANCOVA effect sizes, with small, medium, and large effect sizes interpreted as η^2^ of 0.010–0.058, 0.059–0.13, and ≥ 0.14 respectively [23], and achieved power was computed using G*Power (V3.1.9.2). Spearman correlation coefficients (ρ) were computed to assess relationships between measures of task fatigability with a) perceptions of everyday fatigability, b) pre-fatigue grip strength, and c) pre-fatigue touch-pressure sensitivity. Weak, moderate, and strong correlations were interpreted as ρ of 0.30–0.49, 0.50–0.69, and ≥ 0.70 respectively. Correlation analyses were conducted with Age pooled, and separately for young and older adults.

## Results

Individual values for all measures are included as (S1 Table).

### Age ANCOVAs

#### Anthropometrics

Group means are presented in Table 1. Our sample included 17 young (9 women aged 24.7 ± 3.0 years and 8 men aged 25.0 ± 2.8 years) and 13 old (10 women aged 74.1 ± 8.0 and 3 men aged 73.0 ± 5.6 years) healthy adults. Compared to young adults, old adults were significantly shorter (older group: 160.4 ± 10.4 cm, younger group: 176.6 ± 10.8 cm, F(1,28) = 18.08, p < 0.001, η^2^ = 0.40, power = 0.99) and had higher BMI (older group: 28.4 ± 4.5 kg/m^2^, younger group: 22.1 ± 2.2 kg/m^2^, F(1,28) = 24.1, p < 0.001, η^2^ = 0.47, power = 1.00), but had no difference in mass (older group: 70.8 ± 11.9 kg, younger group: 69.6 ± 12.9 kg, F(28) = 0.80, p = 0.380, η^2^ = 0.029, power = 0.15). Due to the age difference in BMI (in which height was a factor), all further ANCOVA models were also run with both gender and BMI as covariates.

**Table 1:** Demographic and anthropometric means (SD) for young and old adults. Statistically significant age effects (p-values) are also displayed.

Variable	Young (N = 17)	Old (N = 13)	p-value
Number of females	9	10	
Age (years)	24.5 (2.8)	73.8 (7.3)	
Height (cm)	160.4 (10.4)	176.5 (10.8)	p < 0.001
Mass (kg)	70.8 (11.9)	69.6 (12.9)	p = 0.380
Body mass index (kg/m^2^)	28.4 (4.5)	22.1 (2.2)	p < 0.001

#### Everyday perceptions of fatigability

**Fig 1:**
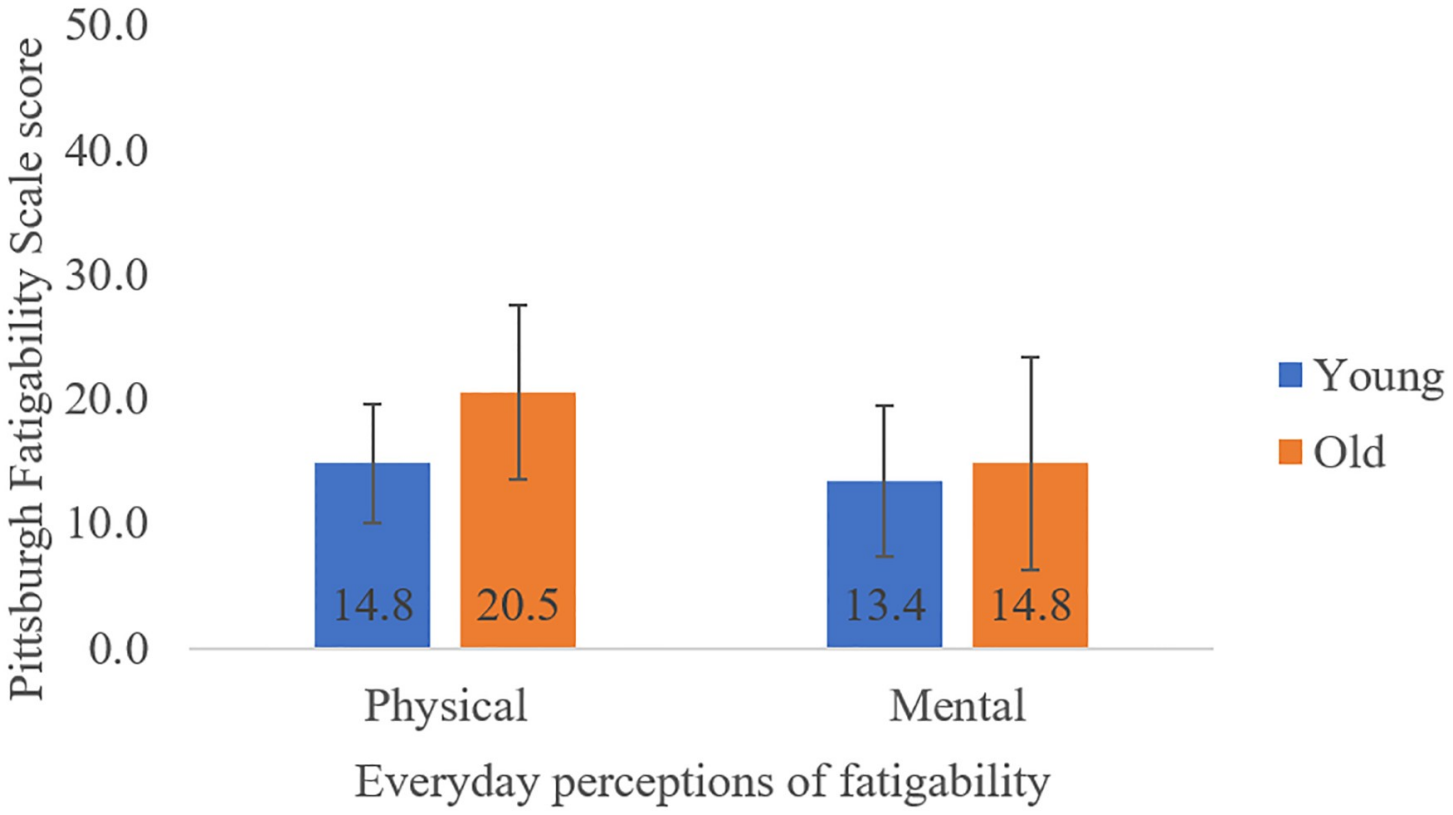
Mean (SD) everyday perceptions of physical and mental fatigability on the Pittsburgh Fatigability Scale, displayed for young and older adults.

Mean scores for each age group are displayed in Fig 1. With gender as a covariate, there were no significant Age differences in PF (older group: 20.5 ± 7.0, younger group: 14.8 ± 4.8) [F(1,27) = 3.29, p = 0.081, η^2^ = 0.11, power = 0.11] or MF (older group: 14.8 ± 8.6, younger group: 13.4 ± 6.0) [F(1,27) = 0.003, p = 0.960, η^2^ < 0.001, power = 0.05]. However, with both gender and BMI as covariates, older adults had significantly higher PF than young adults [F(1,27) = 4.84, p = 0.037, η^2^ = 0.16, power = 0.63]. One participant was excluded from these analyses because they completed only half of the questionnaire in error.

#### Task fatigability

Three of the 13 older and nine of the 17 younger participants reached the 45-minute task termination criteria before reaching the RPE ≥ 8 criteria. However, time to task termination was not significantly different between age groups (older group: 22.85 ± 14.83 min, younger group: 33.76 ± 13.99 min) [F(1,28) = 2.45, p = 0.130, η^2^ = 0.086, power = 0.37] and time to RPE of 5 was not significantly different between age groups (older group: 14.54 ± 12.60 min, younger group: 17.47 ± 13.70) [F(1,28) = 0.50, p = 0.490, η^2^ = 0.019, power = 0.11].

### Age*Condition ANCOVAs

#### Grip strength

**Fig 2:**
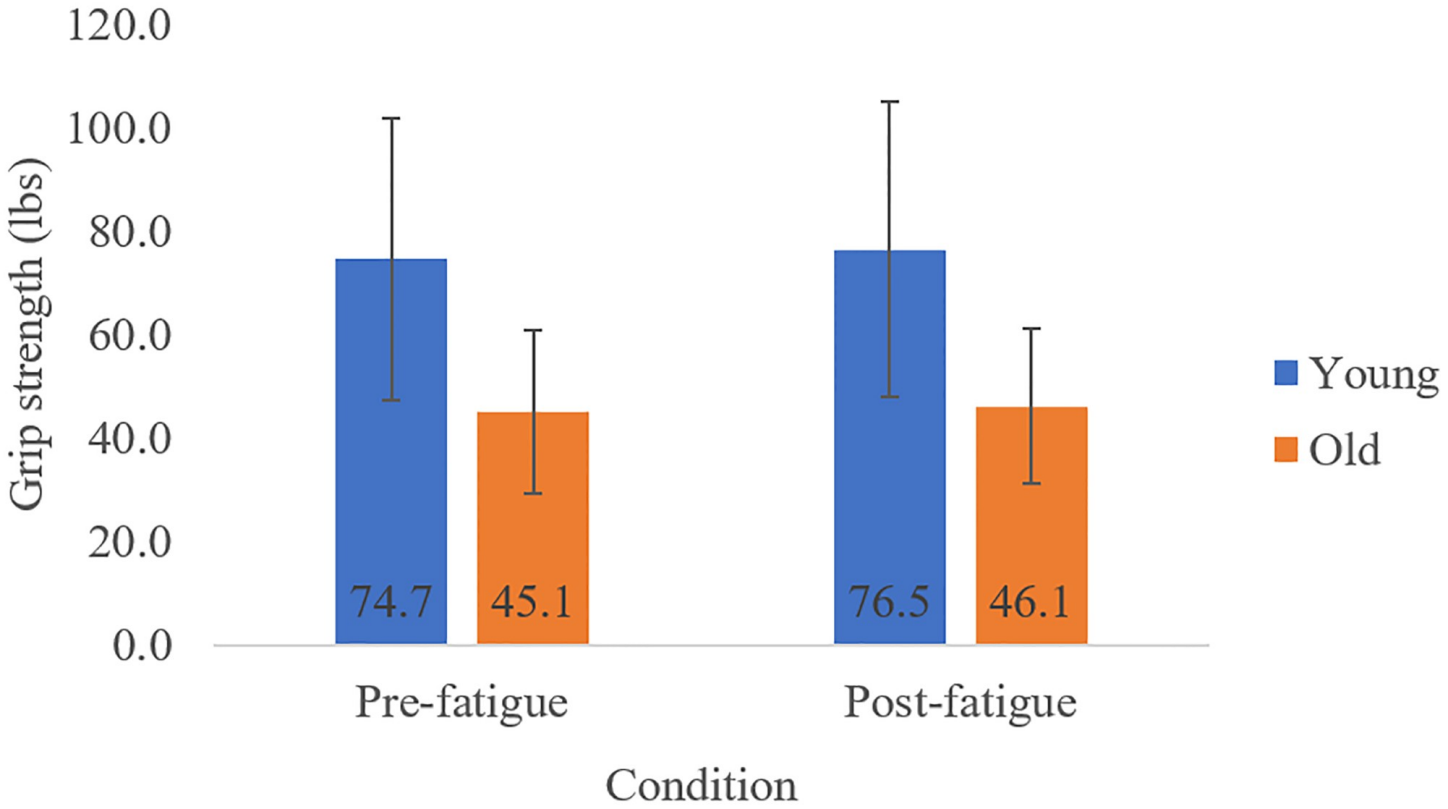
Mean (SD) grip strength (lbs) measured pre and post fatigue, displayed for young and older adults.

Covarying for gender, there was a significant main effect of Age (Fig 2) where older adults (M = 50.50 lb, SD = 15.45 lb) displayed lower grip strength [F(1,26) = 18.02, p < 0.001, η^2^ = 0.40, power = 0.99] compared to young adults (M = 71.46 lb, SD = 27.99 lb), and a main effect of Condition [F(1,26) = 6.84, p = 0.014, η^2^ = 0.20, power = 0.75] where grip strength increased post-fatigue (M = 62.08 lb, SD = 21.82 lb) from pre-fatigue (M = 59.88 lb, SD = 21.62 lb). With gender and BMI as covariates, there was still a significant Age effect [F(1,26) = 22.20, p < 0.001, η^2^ = 0.46, power = 1.00] but no effect of Condition [F(1,26) = 0.040, p = 0.843, η^2^ = 0.002, power = 0.06]. There was no interaction effect in either model.

#### Touch-pressure sensitivity

**Fig 3:**
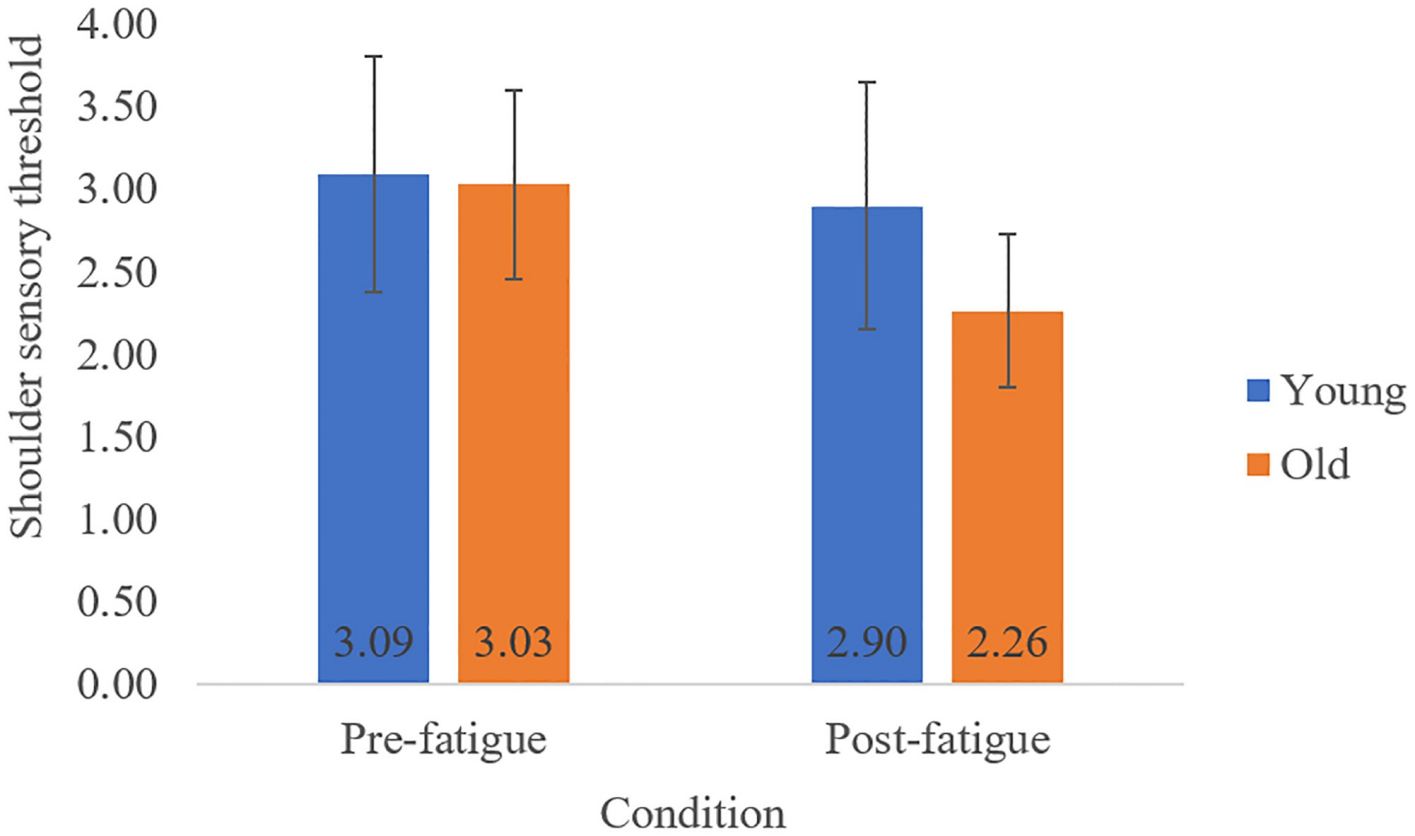
Mean (SD) touch-pressure sensory thresholds at the anterior deltoid (shoulder) for the pre and post-fatiguing task conditions, displayed for young and older adults.

**Fig 4:**
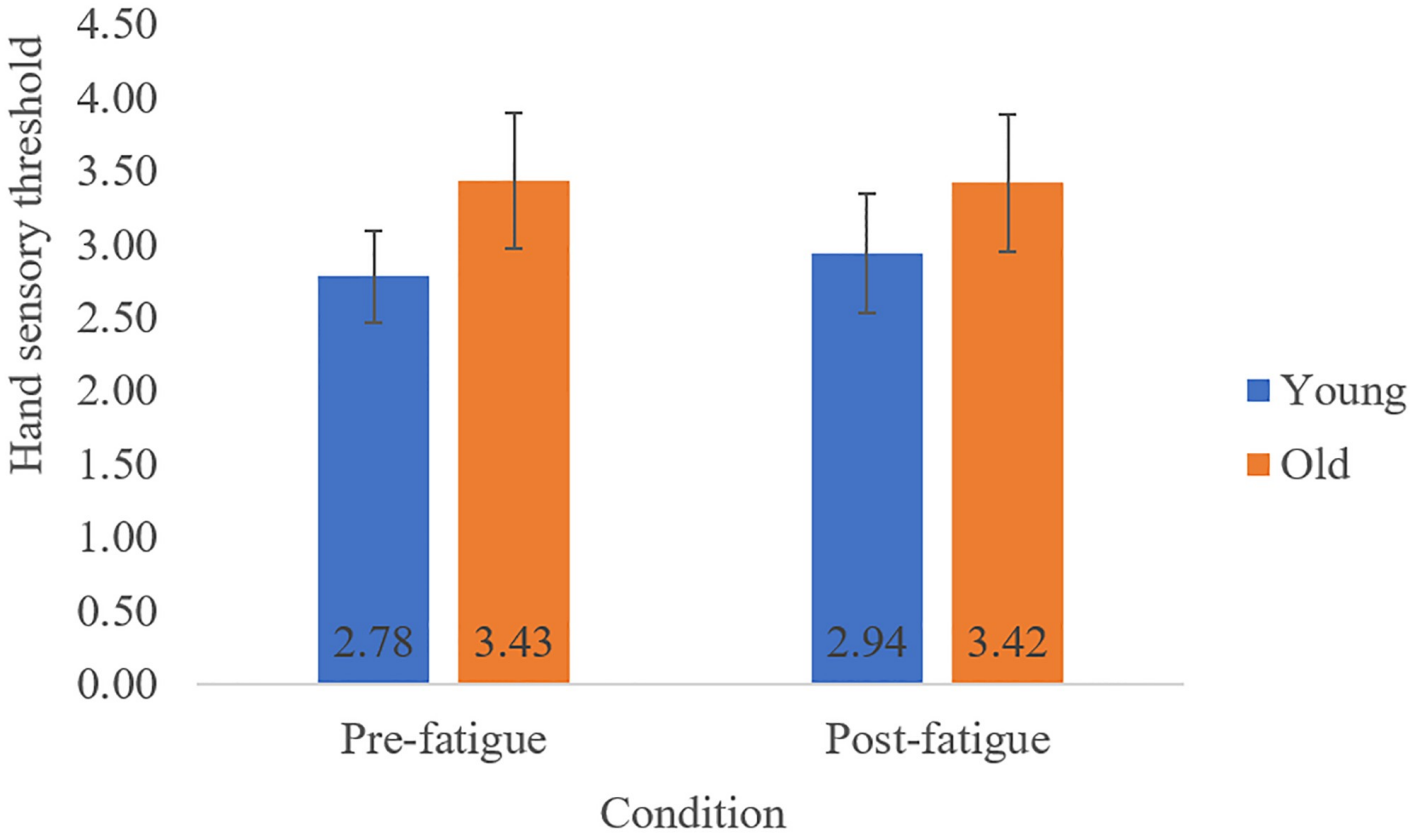
Mean (SD) touch-pressure sensory threshold at the palm of the hand (hand) for the pre and post-fatiguing task conditions, displayed for young and older adults.

There was a significant Age x Condition interaction on QST Shoulder (Fig 3) when covarying for gender [F(1,26) = 8.46, p = 0.007, η^2^ = 0.24, power = 0.84], where older adults displayed decreased sensory thresholds (i.e. increased sensitivity) post-fatigue (M = 2.26, SD = 0.46) from pre-fatigue (M = 3.03, SD = 0.57), but young adults had no change post-fatigue (M = 2.90, SD = 0.75) from pre-fatigue (M = 3.09, SD = 0.71). This interaction was no longer significant, however, when gender and BMI were both covariates [F(1,26) = 1.89, p = 0.180, η^2^ = 0.068, power = 0.27]. There was a main effect of Age on QST Hand (Fig 4) with gender as a covariate [F(1,26) = 12.75, p = 0.001, η^2^ = 0.32, power = 0.95] and with both gender and BMI as covariates [F(1,26) = 10.58, p = 0.003, η^2^ = 0.29, power = 0.92], where older adults (M = 3.42, SD = 0.46) displayed higher sensory thresholds (i.e. lower sensitivity) compared to young adults (M = 2.86, SD = 0.35). No Condition or Age*Condition effects were seen for QST Hand.

### Relationships with task fatigability

Relationships with task fatigability are summarized in Table 2. With Age pooled, longer time to task termination was related to having lower PF (ρ = -0.58, p = 0.001), lower QST Hand (ρ = -0.55, p = 0.002), and higher grip strength pre-fatigue. Longer time to RPE of 5 was also related to having lower PF (ρ = -0.37, p = .049). In the young adult group, task fatigability was not related to pre-fatiguing task measurements of everyday perceptions of fatigability, grip strength, or touch-pressure sensitivity. In the older adult group, longer time to task termination was related to having lower QST Hand pre-fatigue (ρ = -0.56, p = .046).

**Table 2:** Spearman correlation coefficients (p-values) between task fatigability and everyday perceptions of physical fatigability, pre-fatigue grip strength, and pre-fatigue touch-pressure sensitivity. Where PF: physical fatigability, MF: mental fatigability, QST Hand: sensory threshold at the palm of the hand, QST Shoulder: sensory threshold at the anterior deltoid.

	Everyday fatigability		Sensory threshold		Grip strength
Young and old adults	PF	MF	QST Shoulder	QST Hand	
Time to task termination	-.*58 (*.*001)*	-.23 (.23)	.15 (.42)	-.*55 (*.*002)*	.*61 (<* .*001)*
Time to RPE of 5	-.*37 (*.*049)*	-.20 (.29)	-.14 (.45)	-.28 (.14)	.14 (.47)
**Young only**					
Time to task termination	-.39 (.14)	-.20 (.46)	.16 (.55)	-.20 (.43)	.38 (.14)
Time to RPE of 5	-.35 (.19)	-.057 (.83)	-.11 (.67)	.077 (.77)	.021 (.94)
**Old only**					
Time to task termination	-.47 (.10)	-.28 (.35)	.072 (.82)	-.*56 (*.*046)*	.48 (.098)
Time to RPE of 5	-.34 (.26)	-.32 (.29)	-.26 (.40)	-.53 (.061)	.16 (.60)

## Discussion

For the first time, this study investigated how old age affects perceptions of everyday fatigability, grip strength, and touch-pressure sensitivity in a fatiguing upper limb task, and explored relationships in these perceptual, physical, and sensory features of function with task fatigability. No age differences were found in any measure of task fatigability. The main results are 1) old adults had shoulder sensitization with fatigue that was not seen at the hand or in young adults, 2) less task fatigability was associated with lower perceptions of everyday physical fatigability, higher grip strength, and higher touch-pressure sensitivity at the hand, and 3) higher touch-pressure sensitivity at the hand was related to fatigability in old adults but not in young adults. We preface these primary findings by first examining fatigability in the young and old age groups.

### Age-related differences in everyday perceptions of fatigability but not task fatigability

As also reported in our previous study [19], there was an unexpected lack of an age difference in task fatigability. For the age of the older group [10 women (74.1 ± 8.0 years) and 3 men (73.0 ± 5.57 years)], these results reflect a quite high-functioning sample of older adults. This is not overly surprising since older participants were recruited from groups actively engaged in their community, relatively mobile, and not confined to health care facilities. Still, after controlling for the influence of BMI, PF was larger in the older group on average by 5.7 points on the PFS, a large effect size that is also clinically meaningful [17]. These results highlight the discrepancy between perceptions of everyday and task-related fatigability, emphasizing the importance of considering fatigability in both the perceived and performance domains [16].

### Age-related differences in sensorimotor function with and without fatigue

In disagreement with our first hypothesis, we observed decreased QST Shoulder with fatigue in older adults only, indicating an *increase* in touch-pressure sensitivity of the shoulder. Shoulder sensitivity increased in older adults from a pre-fatigue threshold of 3.03, close to the 2.83 threshold deemed as normal [20], to a post-fatigue threshold of 2.26. The change was large in statistical effect size and so we interpret it as a meaningful difference in which BMI was a contributing factor. In contrast, Weber et al [14] reported *decreased* shoulder touch-pressure sensitivity in young adults after repetitive upper limb fatigue. However, like Weber et al, fatigue-related changes in touch-pressure sensitivity were localized to the shoulder and not seen in other locations (e.g. the hand in our study). Although touch-pressure sensitivity did not change in young adults in our study, the combination of these two studies suggests 1) that the fatigue effect on touch-pressure sensitivity is selective to the sites most local to muscle fatigue [14,19], providing no support for central sensitization, and 2) that the sensory response to fatigue changes with old age, potentially with shoulder sensitization in old adults and shoulder desensitization in young adults. Location and age-specific sensitivity to sensory input could be important factors for explaining why fatigue-related motor adaptation strategies differ between old and young adults [19].

In further disagreement with our first hypothesis, we observed no decrease in grip strength with fatigue. This may be task-specific, since the fatiguing task selectively fatigued muscles in the neck and shoulder region [14,19] and likely did not fatigue hand and forearm muscles important for producing gripping force. Accordingly, it is unlikely that any central neural structures were fatigued by our upper limb task. Our finding of lower maximal grip strength in old compared to young adults is in line with previous literature [12,24,25], with this measure being extensively used as a proxy for general physical functioning as well as of impairment [12]. Previous literature suggests that the mechanisms underlying the old age-related decrease in grip strength are related to peripheral changes (e.g. loss of type 2 fibers) [26,27]. Interpreting these grip strength findings with the touch-pressure sensitivity findings described earlier, it appears that fatigue of the neck and shoulder region from a repetitive motion task alters sensory but not physical function in old adults.

### Relationships of perceptual, physical, and sensory function with task fatigability

Pooling across age groups, lower task fatigability was associated with lower PF, higher grip strength, and lower QST Hand (i.e. higher touch-pressure sensitivity at the hand). These relationships come as little surprise with lower perceptions of everyday fatigability, higher grip strength, and higher touch-pressure sensitivity having been previously associated with better functioning [12,15,17,18,20]. Relationships were weak to moderate in size, indicating that the combination of these perceptual, physical, and sensory factors and not one factor alone is likely important in determining an individual’s fatigability to repetitive upper limb motion.

In agreement with our second hypothesis, we found age differences in the relationships with task fatigability. While no relationships were found in the young adult group, lower task fatigability was related to higher hand sensitivity in the old adult group, suggesting that sensory function at the hand may be a more important determinant of fatigability with old age. Reasons for this are not yet clear but having poorer sensory function at the hand may make performing upper limb activities of daily living more challenging, such as household activities captured in the PFS. Indeed, old adults in our sample did have both lower touch-pressure sensitivity at the hand and higher perceptions of everyday fatigability. In turn, those with poorer hand sensation may begin to avoid exercises and activities that require touch-pressure sensitivity at the hand, leading to deconditioning and higher fatigability in experimental tasks such as the one studied in this paper.

### Limitations

This study successfully investigated sensorimotor responses to fatigue and fatigability in young and old adults using simple, affordable, and rigorously validated methods. However, results have some notable limitations. First, we did not consider how sex-based differences in mechanisms of fatigue [28] may have influenced our age-related results. Our sample contained mostly females; while we covaried our statistical models for the influence of sex, investigations of how fatigue develops in aging males and females are needed. Second, our results can only be generalized to healthy adults and the range of ages recruited (55–82 years); clinical conditions related to aging and more advanced may alter findings. Finally, measures obtained are proxies for and not fully representative of perceptual, physical, and sensory function. For instance, we evaluated sensory function by measuring touch-pressure sensitivity but other sensory features (e.g. touch acuity, temperature perception, pressure-pain perception) were beyond the scope of the study, yet could provide further insight on how age affects sensorimotor responses to fatigue.

## Conclusion

To our knowledge, this is the first study to investigate how healthy older age affects touch-pressure sensitivity and grip strength responses to fatigue from repetitive upper limb motion and their relation to fatigability. Fatigue did not affect grip strength but led to increased touch-pressure sensitivity at the shoulder in old adults that was not observed at the hand or in young adults. Adults who had lower task fatigability had a lower perception of everyday physical fatigability, higher grip strength, and higher touch-pressure sensitivity at the hand, with this sensory feature being associated with fatigability in old but not in young adults. With old age, fatigue from repetitive upper limb motion appears to affect sensory function local to the site of muscle fatigue, but not physical function. This sensory function seems to become a more important factor in upper limb fatigability with older age.

## Supporting information

S1 Table Individual values for all demographic, fatigability, grip strength, and touch-pressure sensory threshold measures. (DOCX)
