# Supplementary material for: Effects of old age on fatigability and sensorimotor characteristics of a repetitive upper limb fatiguing task
Source: PLoS One. 2020 Jul 9;15(7):e0235314. doi: 10.1371/journal.pone.0235314 (PMC7347145; doi:10.1371/journal.pone.0235314)
Supplement: S1 Table — (DOCX) [file pone.0235314.s001.docx]

Supplemental Table 1. Individual values for all demographic, fatigability, grip strength, and touch-pressure sensory threshold measures.

| Participant | Demographics | | | | | Everyday perceptions of fatigability | | Task fatigability | | Grip strength (lbs) | | Touch-pressure sensory threshold | | | |
| --- | --- | --- | --- | --- | --- | --- | --- | --- | --- | --- | --- | --- | --- | --- | --- |
|  | Gender | Age (years) | Height (cm) | Mass (kg) | BMI (kg/m^2^) | Physical | Mental | Time to task termination (min) | Time to RPE of 5 (min) | Pre-fatigue | Post-fatigue | Anterior deltoid; Pre-fatigue | Anterior deltoid; Post-fatigue | Hand; Pre-fatigue | Hand; Post-fatigue |
| 1Y | F | 28 | 183.5 | 66.6 | 19.8 | 15 | 20 | 45 | 42 | 63.0 | 58.6 | 3.61 | 3.84 | 3.22 | 3.84 |
| 2Y | F | 28 | 172.0 | 75.6 | 25.6 | 15 | 10 | 45 | 41 | 48.0 | 54.0 | 1.65 | 1.65 | 2.44 | 2.83 |
| 3Y | F | 24 | 180.0 | 66.5 | 20.5 | 16 | 9 | 5 | 3 | 53.7 | 52.4 | 3.61 | 3.61 | 2.83 | 2.83 |
| 4Y | F | 28 | 156.0 | 43.1 | 17.7 | 24 | 11 | 17 | 8 | 39.5 | 40.0 | 4.08 | 3.61 | 2.83 | 2.83 |
| 5Y | F | 22 | 164.0 | 55.1 | 20.5 | 9 | 6 | 13 | 12 | 42.8 | 42.2 | 2.44 | 4.08 | 2.83 | 2.83 |
| 6Y | M | 22 | 186.0 | 76.7 | 22.2 | 14 | 14 | 45 | 39 | 88.3 | 91.7 | 3.61 | 2.44 | 2.44 | 2.36 |
| 7Y | M | 25 | 184.5 | 80.7 | 23.7 | 11 | 4 | 45 | 20 | 98.6 | 106.9 | 3.22 | 2.83 | 2.44 | 2.83 |
| 8Y | F | 21 | 165.5 | 53.2 | 19.4 | 17 | 18 | 45 | 15 | 45.5 | 47.9 | 2.83 | 2.83 | 2.83 | 2.83 |
| 9Y | F | 22 | 168.5 | 69.7 | 24.6 | 9 | 10 | 30 | 13 | 64.5 | 65.9 | 2.83 | 2.83 | 2.44 | 2.44 |
| 10Y | M | 24 | 181.0 | 71.7 | 21.9 | 15 | 13 | 45 | 6 | 95.9 | 105.5 | 3.22 | 2.36 | 2.36 | 2.44 |
| 11Y | M | 28 | 176.5 | 69.0 | 22.2 | 7 | 11 | 45 | 9 | 79.2 | 80.4 | 3.61 | 2.83 | 2.83 | 3.61 |
| 12Y | F | 22 | 170.3 | 62.6 | 21.6 | 19 | 23 | 32 | 17 | 58.6 | 58.8 | 3.84 | 2.44 | 3.22 | 3.61 |
| 13Y | M | 22 | 191.5 | 91.1 | 24.8 | 19 | 19 | 30 | 5 | 113.6 | 116.4 | 2.83 | 3.61 | 2.44 | 2.83 |
| 14Y | M | 30 | 187.5 | 86.2 | 24.5 | 22 | 20 | 28 | 9 | 116.8 | 126.0 | 2.83 | 2.44 | 2.83 | 2.83 |
| 15Y | M | 25 | 171.5 | 60.4 | 20.5 | 18 | 15 | 45 | 10 | 85.6 | 96.6 | 3.84 | 3.84 | 3.22 | 2.83 |
| 16Y | M | 24 | 195.5 | 88.4 | 23.1 | 9 | 4 | 45 | 39 | 120.9 | 106.4 | 2.83 | 2.36 | 2.83 | 2.83 |
| 17Y | F | 22 | 167.5 | 66.8 | 23.8 | 13 | 21 | 14 | 9 | 54.6 | 51.0 | 1.65 | 1.65 | 3.22 | 2.83 |
| 1A | F | 72 | 160.5 | 82.3 | 32.0 | 15 | 10 | 16 | 10 | 39.4 | 43.6 | 3.61 | 2.83 | 3.61 | 3.61 |
| 2A | F | 77 | 159.0 | 56.1 | 22.2 | 29 | 26 | 25 | 16 | 38.7 | 41.1 | 2.83 | 2.44 | 3.22 | 3.84 |
| 3A | M | 78 | 185.0 | 78.2 | 22.9 | 18 | 8 | 20 | 12 | 75.1 | 77.8 | 3.22 | 2.83 | 2.83 | 3.22 |
| 4A | F | 79 | 150.0 | 52.0 | 23.1 | 12 | N/A | 45 | 39 | 40.4 | 43.9 | 3.22 | 2.83 | 2.83 | 2.83 |
| SA | M | 74 | 160.0 | 67.6 | 26.4 | 20 | 16 | 6 | 3 | 39.5 | 37.3 | 2.83 | 1.65 | 3.61 | 3.61 |
| 6A | F | 72 | 151.5 | 75.0 | 32.7 | 31 | 20 | 6 | 3 | 40.0 | 45.7 | 3.22 | 2.36 | 3.84 | 3.61 |
| 7A | F | 81 | 153.5 | 72.1 | 30.6 | 17 | 10 | 4 | 3 | 30.6 | 32.4 | 2.83 | 2.36 | 3.22 | 3.61 |
| 8A | F | 70 | 151.5 | 68.7 | 29.9 | 27 | 25 | 27 | 21 | 36.4 | 37.3 | 2.44 | 1.65 | 3.61 | 2.83 |
| 9A | F | 80 | 162.0 | 61.9 | 23.6 | 18 | 7 | 18 | 12 | 25.4 | 23.8 | 1.65 | 1.65 | 3.61 | 3.61 |
| 10A | F | 82 | 156.0 | 70.2 | 28.9 | 22 | 18 | 28 | 16 | 43.6 | 46.0 | 3.22 | 2.44 | 3.61 | 3.84 |
| 11A | F | 73 | 153.0 | 64.8 | 27.7 | 29 | 28 | 12 | 6 | 42.6 | 45.2 | 3.61 | 2.36 | 4.08 | 3.61 |
| 12A | M | 67 | 176.0 | 72.6 | 33.8 | 21 | 8 | 45 | 1 | 79.9 | 74.0 | 3.84 | 1.65 | 3.61 | 3.84 |
| 13A | F | 55 | 167.5 | 99.1 | 35.3 | 8 | 2 | 45 | 41 | 54.7 | 51.8 | 2.83 | 2.36 | 2.36 | 2.36 |
